# Epidemiology, Causes, and Management of Gastro-esophageal Reflux Disease: A Systematic Review

**DOI:** 10.7759/cureus.47420

**Published:** 2023-10-21

**Authors:** Tariq M Shaqran, Mustafa M Ismaeel, Aljoharh Abdulaziz Alnuaman, Fatimah A Al Ahmad, Ghadeer A Albalawi, Jori N Almubarak, Rakan S AlHarbi, Rayan S Alaqidi, Yaqin A AlAli, Khaled S Alfawaz, Abdulrahman A Daghreeri

**Affiliations:** 1 Family Medicine, King Salman Armed Forces Hospital, Tabuk, SAU; 2 Medicine, Cairo University, Cairo, EGY; 3 Pediatrics, Faculty of Medicine, University of Tabuk, Tabuk, SAU; 4 Medicine, Imam Abdulrahman Bin Faisal University, Dammam, SAU; 5 Medicine, King Khalid Military City Hospital, Hafar Al Batin, SAU; 6 Medicine, Salmaniya Medical Complex, Manama, BHR; 7 Internal Medicine, Vision College, Riyadh, SAU; 8 Medicine, King Faisal University, Ahsa, SAU; 9 Medicine, Faculty of Medicine, King Abdulaziz University, Jeddah, SAU; 10 Medicine, Jazan University, Jazan, SAU

**Keywords:** acid reflux, proton-pump inhibitor, management, diagnosis, gastro-esophageal reflux disease

## Abstract

Our comprehensive systematic review aimed to examine gastroesophageal reflux disease (GERD), a disorder that occurs when stomach contents flow back into the esophagus. It may manifest as either non-erosive reflux disease or erosive esophagitis. The activity depicts the assessment and medical management of GERD and emphasizes the interprofessional team's involvement to enhance care for people with this ailment. Data sources were PubMed/Medline and Embase. Our review investigated English-language articles (from 2014 to 2023) according to the Preferred Reporting Items for Systematic Reviews and Meta-Analyses (PRISMA) guidelines. Overall, there were seven articles. Surveys and analyses of national databases were the most widely used methods (n=7). The search identified 3,730 studies, and seven were eligible for inclusion in the analysis. Further understanding of GERD and treatment protocols may help improve evaluation and management in the future. Millions of individuals worldwide suffer from GERD, a common clinical condition. Patients can be identified by symptoms that are both common and uncommon. For many GERD patients, acid suppression treatment reduces symptoms and avoids clinical complications. Our capacity to recognize and treat disease consequences has improved with the advancement of diagnostic and treatment methods. Here, we go into the etiology and consequences of GERD and offer details on the treatment strategy for this prevalent illness.

## Introduction and background

Gastroesophageal reflux disease (GERD) is a chronic gastrointestinal disorder that leads to the regurgitation of gastric contents into the esophagus. With a prevalence of 20% in the US, GERD significantly impacts the economy and quality of life. GERD can be triggered by intrinsic and/or structural mechanisms that break the barrier of esophagogastric junctions, the esophagus exposed to acidic contents of the stomach. Heartburn, regurgitation, chest discomfort, tooth erosions, persistent cough, asthma, and laryngitis are all symptoms [1].

GERD can be classified into three main categories: the first one is non-erosive reflux disease (NERD), the second is erosive esophagitis (EE), and the last one is Barrett esophagus (BE). The most prevalent condition is NERD, while EE and BE occur in 30% and between 6% and 12% of patients, respectively. While lifestyle changes and medications from the proton pump inhibitor (PPI) family are commonly used to treat GERD, medically resistant GERD has become more prevalent [2].

GERD has no known etiology, although various risk factors were discovered, including motor abnormalities like esophageal dysmotility, decreased esophageal acid clearance, and reduced the tone of lower esophageal sphincter. Anatomical factors such as hiatal hernia and increased intra-abdominal pressure in obesity increase the likelihood of getting GERD. Obesity is also associated with a higher risk of developing symptoms related to GERD, EE, and esophageal cancer [3].

Malfertheiner et al. discovered that BMI increases the odds ratio (OR) for erosive reflux disease in over 6,000 participants in their ProGERD trial. Other risk factors include age, low socioeconomic status, tobacco use, alcohol consumption, connective tissue disorders, pregnancy, postprandial supination, and various drugs such as anticholinergics, benzodiazepines, and NSAIDs [4].

GERD is a common gastrointestinal disorder, affecting around 20% of adults in Western cultures. The prevalence in the US ranges from 18.1% to 27.8%, with a slightly higher rate in men. A meta-analysis found that women have a marginally higher prevalence of GERD symptoms compared to men. Women with GERD symptoms are more likely to have NERD than men with EE. Men with long-standing GERD symptoms are more likely to develop Barrett's esophagus [5].

The influence of the LES's tone, the existence of a hiatal hernia, the esophageal mucosal defense against refluxate, and esophageal motility are among the factors that best explain the multifaceted pathophysiology of GERD. However, patients with GERD may experience frequent transient LES relaxations (TLESRs), exceeding intragastric pressure more than LES pressures, leading to reflux. Alcohol usage, smoking, caffeine, pregnancy, and some medicines such as calcium channel blockers are all factors affecting the tone of the LES and TLESRs [6].

Hiatal hernia is often linked to GERD, as it has an effect on the LES's function. Patients with GERD exhibit identical LES defects and acid clearance, regardless of hernia size. However, large hernias cause the LES to be shorter and weaker, resulting in more reflux episodes. Ott et al. discovered that 94% of individuals with reflux esophagitis have a hiatal hernia [7].

The esophageal mucosa acts as a protective barrier against GERD substances, but prolonged exposure to refluxate can damage it. Delayed gastric emptying is thought to contribute to GERD symptoms because of gastric distention and greater exposure to refluxation. It is uncertain how gastroparesis affects GERD [8]. Diener et al. discovered that 21% of GERD patients have reduced esophageal peristalsis, resulting in poor gastric reflux clearance, acute symptoms of reflux, and mucosal injury [9].

The goal of GERD care is to alleviate symptoms and avoid complications, such as esophagitis, BE, and cancer of the esophagus. Lifestyle changes, pharmacological care such as (antacids and antisecretory medications), surgical treatments, and endoluminal therapies are all alternatives for treatment. Weight reduction, avoiding foods before night, and keeping excellent sleep hygiene are all examples of lifestyle changes. Elevating the head of the bed has been proven to relieve GERD symptoms and enhance pH monitoring. Dietary changes that include the avoidance of chocolate, coffee, spicy meals, citrus, and soft drinks are contentious and are not generally suggested by current ACG recommendations [10].

Medical therapy, which includes antacids, antisecretory drugs such as histamine receptor antagonists (H2RAs), and prokinetic medications, is used for individuals who do not react to lifestyle changes. In the United States, there are two drugs accepted by the FDA H2RAs accessible over the counter (famotidine and cimetidine), while ranitidine and nizatidine have been recalled due to potential health risks. There are six PPIs accessible in the US, three of which are over-the-counter and three of which are prescription-only. PPI treatment is seen to be the best option when treating GERD, with improved symptom management, healing of the underlying esophagitis, and lower recurrence rates. PPI medication should be started once a day prior to the first meal, with bedtime dosing for evening symptoms, according to ACG recommendations. Due to a lack of evidence and potential side effects on the nervous and circulatory systems, the use of prokinetic medicines such as metoclopramide and domperidone for GERD is restricted [10].

Patients with GERD who are medically resistant and have noncompliance, adverse effects, a significant hiatal hernia, or discontinued long-term medication may consider surgical surgery. In obese individuals, alternatives include laparoscopic Nissen fundoplication, 180° LAF, and bariatric surgery. Due to the increased rate of fatness in the US, a gastric bypass operation is becoming a popular therapy, particularly for obese patients with GERD [11].

Before surgical treatment, the American College of Gastroenterology (ACG) suggests preoperative pH monitoring and esophageal manometry procedures to exclude achalasia or undetected scleroderma-like esophagus. Two meta-analyses produced conflicting findings, with one showing enhancement of GERD symptoms following surgery and the other indicating ambiguity about surgical benefits. Patients having fundoplication may experience postoperative complications such as bloating, dysphagia, and belching. Roux-en-Y gastric bypass (RYGB) is an effective treatment for GERD symptoms and is indicated for severe GERD patients prior to surgery [12].

Endoscopic therapies for GERD management have been developed using minimally invasive techniques. However, many have been discontinued due to a lack of long-term efficacy. Magnetic sphincter augmentation (MSA) and transoral incisionless fundoplication (TIF) with EsophyX (EndoGastric Solutions, Redmond, WA) are two current endoluminal procedures. A meta-analysis found that TIF 2.0 patients had higher esophageal pH, lower PPIs, and higher quality of life. Another study found TIF in combination with EsophyX to be a good long-term therapy option for symptomatic GERD with hiatus hernia. A meta-analysis of Nissen fundoplication and MSA revealed that MSA is a successful GERD treatment option [13].

The aims of GERD management are to alleviate symptoms and avoid complications such as esophagitis, BE, and cancer of the esophagus. Lifestyle changes, pharmacological care such as antacids and antisecretory medications, surgical treatments, and endoluminal therapies are all alternatives for treatment.

## Review

Methods

According to the Preferred Reporting Items for Systematic Reviews and Meta-Analyses (PRISMA) 2020 standards, the procedures for the current systemic were developed.

Search

**Figure 1:**
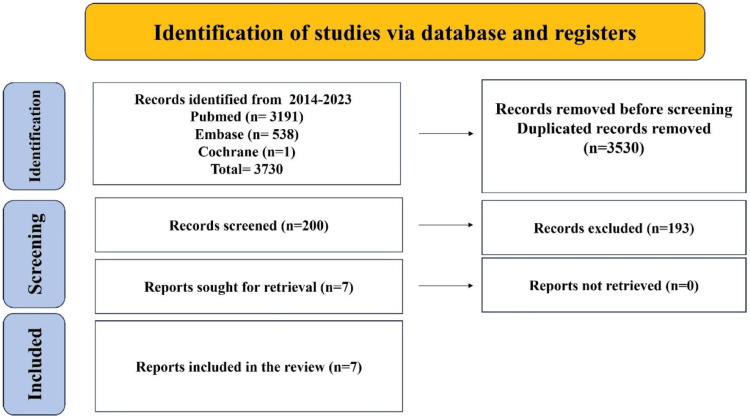
Study flow diagram of Preferred Reporting Items for Systematic Reviews and Meta-Analyses (PRISMA)

For this review, keywords and medical subjects headings phrases were used to search PubMed/Medline (2014-August 2023) and Embase (2014-August 2023) for three important concepts: gastro-esophageal reflux disease, diagnosis and management, and proton pump inhibitors (PPIs). Limits were applied so that only English-language items were included. The supplementary material contains a detailed discussion of the PubMed search methodology. Examining the references found in the identified papers led to the creation of additional studies. After retrieving all full texts, seven articles were discovered to be part of this review. The PRISMA technique was used for search screening and article shortlisting (Figure 1).

Inclusion criteria: English articles of newly diagnosed cases of uncomplicated gastroesophageal reflux disease with hiatus hernia patients (aged between 20 and 60 years) with symptoms of gastroesophageal reflux disease whose diagnosis has been confirmed by endoscopy and manometry.

Exclusion criteria: Presence of comorbid conditions such as hypertension and diabetes mellitus as well as pregnancy, incomplete data, and research data before 2010 were excluded.

Primary Outcomes

The primary outcome was the management of gastro-esophageal reflux disease.

Data Collection

A total of 200 papers were included after titles and abstracts were checked out of the 3,730 studies that were found through the search. Seven papers were qualified for analysis after full-text reading (Figure 1).

Data Extraction

Table 1 provides a brief description of the data, which include the author(s) name and year, the study aims, and finally the conclusion of the study.

**Table 1:** Brief description of the data GERD: Gastroesophageal reflux disease; PPI: Proton-pump inhibitor; SDM: Self-directed management; PRO's: Patient-Reported Outcomes; NERD: Non-erosive reflex disease

Serial no.	Author and year	Study design	Treatment	Outcome
1	Frazzoni et al. 2023 [14]	Practice-oriented concise answers to clinical questions, with the aim of optimizing patient GERD management and healthcare resource use	Patients were diagnosed by endoscopy and treated with proton-pump inhibitors and surgery.	Only in cases where eosinophilic esophagitis is suspected should esophageal biopsies be carried out. Repeat endoscopy should only be performed on patients with LA grade C and D esophagitis following PPI medication. The preferred diagnostic for confirming or ruling out GERD is impedance-pH monitoring. Patients with GERD who also experience concurrent dyspeptic symptoms may benefit from prokinetics.
2	Klenzak et al. 2018 [15]	Management of gastroesophageal reflux disease: using endoscope versus wireless pH probe	In ambulatory 24-hour pH monitoring, wireless pH sensors are used to quantify the amount of gastric acid that is directly exposed to the esophagus.	With the use of this diagnostic technique, reflux frequency may be measured and information on the correlation between the onset of symptoms and reflux episodes can be obtained.
3	Shah et al. 2014 [16]	Management of gastroesophageal reflux disease: a review of medical and surgical management	For the study, 50 individuals with confirmed endoscopic and esophageal manometry diagnoses of gastroesophageal reflux disease were selected. Over the course of three months, the patient was prescribed a proton pump inhibitor (tablet pantoprazole, 40 mg twice a day) and a prokinetic drug (tablet levosulpiride, 75 mg twice a day).	After three months, twenty patients had improved symptoms, and thirty had not. According to the study, all patients with reflux should follow a conservative treatment plan; if non-invasive measures are not successful, surgery can be required. Laparoscopy Toupet's fundoplication is a minimally invasive surgical treatment option that works well, but more research with a bigger sample size and longer follow-up is needed to determine its long-term consequences.
4	Roark al. 2020 [17]	Management of gastroesophageal reflux disease via various methods in a comprehensive review	Among the various management options include surgery, minimally invasive procedures, medication therapy, and lifestyle modification. The ultimate decision about treatment should be based on a case-by-case, multidisciplinary team that includes a surgeon, gastroenterologist, and primary care physician, using an individual, patient-centered approach.	The ultimate decision about treatment should be based on a case-by-case, multidisciplinary team that includes a surgeon, gastroenterologist, and primary care physician, using an individual, patient-centered approach.
5	Chhabra et al. 2022 [18]	Gastroesophageal reflux disease (GERD): highlighting lifestyle changes	Lately, the side effects of the PPI class of drugs have come to the attention of both patients and clinicians. Furthermore, there has been a notable decline in surgical fundoplication and an increase in the development of non-medical therapeutic approaches for GERD. Making lifestyle adjustments is essential for the treatment of GERD.	There are individual differences in how GERD symptoms react to various diets. The research suggests that there might be a link between the incidence of reflux and chocolates, salty foods, foods high in fat, and aerated drinks, even if there is not enough evidence to back up this claim. Other factors in lifestyle adjustments include smoking, being overweight or obese, the head of the bed, patients' laying down positions, and physical activity.
6	Savarino et al. 2021 [19]	Pharmacological management of gastro-esophageal reflux disease collected from different reviews	This review offers an overview of the most recent developments in the pathophysiology of this illness and the novel medications that have entered the market to fill in some of the gaps left by the failure of PPIs in standard therapy. Diagnostic tests that help differentiate between esophageal functional problems and real GERD include high-resolution manometry and 24-hour impedance-pH monitoring.	The main treatment for this condition is proton pump inhibitors (PPIs), although some people still have symptoms. Patients with non-erosive reflux disease (NERD) encounter difficult clinical circumstances that call for testing and steer clear of empirical therapy. Novel medications have been created to strengthen the protective qualities of the mucosa, and randomized clinical trials have shown encouraging outcomes.
7	Katzka et al. 2020 [20]	Advances in the diagnosis and management of gastroesophageal reflux disease	The goals of care are to reduce potential health hazards and relieve symptoms by using a mix of surgery, medication (mostly to prevent or control stomach acid secretion), lifestyle changes, and diagnostic tests.	The goals of management include reducing health risks and relieving symptoms by lifestyle changes, medication, surgery, and diagnostic testing. However, GERD is a chronic, recurrent illness; thus, it is debatable if this applies to different GERD subtypes.

Discussion

GERD is a prevalent condition with no strong association with symptoms. Demographic factors such as obesity and tobacco use are strongly associated with symptoms and complications. Classic symptoms such as pyrosis and regurgitation are only somewhat sensitive. GERD and other conditions such as gastroparesis, non-ulcer dyspepsia, and eosinophilic esophagitis have significant overlap, posing management dilemmas [21].

GERD is a frequent digestive illness around the world, with a prevalence range of 18.1%-27.8% in North America. It is characterized by symptoms and complications resulting from stomach contents refluxing into the esophagus. Diagnosis is based on classic symptoms and acid suppression response. GERD is a significant health concern, affecting quality of life (QoL) and morbidity. Successful treatment improves QoL, physical pain, vitality, and emotional well-being. However, the cost of treating GERD patients is twofold more than those without GERD. Heartburn is the most common symptom of GERD, a burning sensation in the chest and mouth caused by acid reflux into the esophagus. It is frequently accompanied by a sour aftertaste in the lower part of the mouth [22].

GERD is a prevalent cause of non-cardiac pain in the chest, and distinguishing between the underlying reason and the symptoms is critical. A favorable clinical history may point to GERD as a possible reason. Reflux toward the larynx is more likely to cause extraesophageal symptoms such as throat clearing and a sore throat. A globus feeling may also occur in patients, which is thought to be produced by acid exposure in the hypopharynx. Acid reflux can induce bronchospasm, exacerbation of asthma, coughing, dyspnea, and wheezing. Chronic feelings of nausea and vomiting can happen in certain patients with GERD [23].

Untreated GERD can progress to serious consequences, such as esophagitis and Barrett's esophagus, which cause esophageal erosions, ulcerations, and constriction. Severe cases may cause gastrointestinal bleeding, while chronic inflammation from acid exposure can cause scarring and peptic strictures, often causing dysphagia [24].

Persistent acid reflux can lead to Barrett's esophagus, an intestinal metaplasia resulting from acid exposure. This disorder causes the typical squamous cell epithelium to be replaced with columnar epithelium, including goblet cells, which can develop into esophageal adenocarcinoma; hence, early discovery is critical for prevention and care [25]. GERD is clinically diagnosed with characteristic symptoms and a reaction to acid suppression, which is frequently prompted by heartburn. If patients respond to empiric therapy without alarm characteristics or symptoms, no additional workup is required [26].

This review highlights the surgical management of GERD to find the more appropriate management options. This systematic review identified seven studies presenting the in-hospital medical response to GERD. In the US, GERD is a popular gastrointestinal condition, and it is a major cause of morbidity and mortality. Advanced age, male gender, white race, weight gain in the abdomen, and cigarette use are all risk factors. Heartburn and regurgitation have been frequent symptoms of GERD, with coexisting dysphagia being a concerning indication. The complexity of GERD coincides on top of other illnesses, making patient treatment more difficult [27].

With regional fluctuation, the overall frequency of GERD symptoms is roughly 13%. With nearly 25%, South Asia and Southeast Europe have the highest incidence, whereas the lowest is found in the Southeast Asian region, Canada, and France. There is no information on the frequency of GERD in Africa. The prevalence range in the US is 6%-30%, with variation depending on the questionnaire utilized. In the US, the rate of GERD symptoms is over 20%, with 110,000 hospitalizations each year. Since the early to mid-1990s, the frequency of symptoms of GERD in the US, Europe, and Southeast Asian regions has grown by 50%. A longitudinal research in a Norwegian county discovered that the yearly incidence of new symptoms of GERD was 3.1%, with severe GERD symptoms occurring in 0.2% of the population. Symptoms reduced spontaneously in 2.3% of people with GERD at the start and 1.2% of those with acute GERD [5].

GERD patients must be evaluated for alarm features, prompting urgent endoscopic evaluation. If no warning symptoms are present, the first treatment should focus on lifestyle modification. Although most research on lifestyle and dietary modifications in GERD are insufficiently powered, lifestyle changes remain the primary goal for symptom reduction and improved QoL in GERD management [28].

GERD is typically managed through PPI therapy and lifestyle changes. For non-alert patients, PPI therapy can be optimized or increased to twice daily. For persistent symptoms, endoscopy and esophageal physiology tests can be used. Endoscopic therapies such as laparoscopic fundoplication and MSA can be beneficial. Neuromodulators or psychological therapies can be used to treat functional illnesses that overlap with or resemble GERD. Potassium-competitive acid inhibitors, reflux-reducing medicines, bile acid binders, injections to the gastro-esophageal junction, and stimulation with electricity of the gastroesophageal sphincter may be used in the future [29].

Pharmacological therapy may have potential adverse events, but further research is needed to determine its existence. Lifestyle changes should be regarded as first-line therapy, with pharmacologic therapy sought if lifestyle changes are inadequate or cannot be implemented. In high-risk individuals, extraesophageal sources of GERD-like symptoms should be explored [30].

GERD is a prevalent disease in the Western world, affecting 10%-20% of the population. Symptoms include acid regurgitation and heartburn, with high specificity for GERD. With these symptoms, a preliminary diagnosis can be formed, but further testing may be necessary to confirm the diagnosis and assess for complications. Complications of GERD involve erosive esophagitis, peptic ulcer, Barrett's esophagus, esophageal cancer, and pulmonary illness. Lifestyle changes, medication, and surgical therapies are all used to manage the condition. Weight reduction and head-of-bed elevation can improve esophageal pH and symptoms. Medical treatment includes acid regulation with antacids, H2-blockers, or PPI. After a pre-operative assessment, certain individuals may require anti-reflux surgery [31].

GERD is a prevalent, chronic disorder with limited knowledge and changing management. Recent advances in disease etiology and novel medications have aided in overcoming traditional therapy's unmet needs. Diagnostic tests, such as 24-hour impedance-pH monitoring and high-resolution manometry, can help differentiate real GERD from esophageal functioning problems. PPIs are the primary therapy for GERD; however, a considerable number of patients continue to have symptoms. These situations mostly affect NERD patients; hence, understanding the causes of PPI failure is critical. The management of these individuals necessitates testing and the avoidance of empiric therapies, which are frequently ineffective, expensive, and possibly harmful. In randomized clinical trials, new medications have shown promising effects [19].

## Conclusions

In the US, the rising obesity epidemic is correlated with an increased prevalence of GERD. Treatment primarily consists of changing one's lifestyle, such as eating habits, weight, and diet. PPIs continue to be the most successful medication for managing symptoms, given the variety of pharmacologic treatments available. Nonetheless, an examination of the proper dosage and possible adverse effects of proton pump inhibitors should be performed prior to writing a prescription. However, further research is needed to determine whether there is a true causative relationship, despite evidence suggesting potential connections with bone mineral disorders, dementia, and renal injury.
